# Covid-19 and College Students: Food Security Status before and after the Onset of a Pandemic

**DOI:** 10.3390/nu13020628

**Published:** 2021-02-15

**Authors:** Kaley Mialki, Lisa A. House, Anne E. Mathews, Karla P. Shelnutt

**Affiliations:** 1 Food Science and Human Nutrition Department, University of Florida, Gainesville, FL 32611, USA; kmialki@ufl.edu (K.M.); anne.mathews@ufl.edu (A.E.M.); 2 Food and Resource Economics Department, University of Florida, Gainesville, FL 32611, USA; lahouse@ufl.edu; 3 Department of Family, Youth and Community Sciences, University of Florida, Gainesville, FL 32611, USA

**Keywords:** food insecurity, college, student, Covid-19, pandemic

## Abstract

While the Covid-19 pandemic has increased the number of food insecure households in the United States (US), it is unclear how it has affected college student food security status. College students are ineligible for many Covid-19-related economic relief programs and may find it even more difficult to cope during the pandemic. Therefore, the purpose of this study was to identify and describe the prevalence of food insecurity at a public university before and after the onset of Covid-19 as well as factors associated with any change in food security. Researchers administered a cross-sectional, non-probability survey to college students (*n* = 3206) that assessed food security status prior to and after the onset of the Covid-19 pandemic, as well as questions related to various sociodemographic characteristics. Data were analyzed using descriptive statistics. Thirty-eight percent of students experienced a change in food security as a result of the pandemic, with 59.6% becoming less food secure, and 40.4% becoming more food secure. Characteristics that were associated with changes in food security included changes in housing and employment status as a result of the pandemic. These findings suggest that the pandemic led to changes in food security among college students, and that economic relief efforts should include college students, who are disproportionately affected by food insecurity.

## 1. Introduction

On 11 February 2020, the World Health Organization named the virus that was first identified in Wuhan China and later spread across the globe [1]. Coronavirus disease 2019, referred to as Covid-19, characterized the novel strain of coronavirus that causes a range of symptoms including fever, chills, cough, shortness of breath, fatigue, headache, loss of taste or smell, sore throat, congestion, nausea, vomiting, and diarrhea [2]. Covid-19 quickly spread to the United States and took a toll on the population’s healthcare, economic, and political systems [3,4,5]. Government and public health agencies have imposed social distancing restrictions and ordered business closures to reduce the spread of the disease. While helping to contain the virus and protect the health of Americans, these changes are challenging Americans financially, and disproportionately affecting Americans who already face social and economic inequalities [6]. 

One cause of financial hardship in the United States (US) is the rise in unemployment. The unemployment rate increased from 4% in March 2020 to 14.7% in April 2020 and has since fallen but not to pre-pandemic levels [7]. Unemployment rates are higher among teenagers, Blacks/African Americans, Asians, and Hispanics/Latinos [7]. The federal government has responded to the spike in unemployment through various measures, including extended deadlines for filing and paying taxes, Economic Impact Payments for some adults and children, and paid sick-leave for workers [8]. In an effort to help families access nutritious foods during the pandemic, the United States Department of Agriculture (USDA) Food and Nutrition Service (FNS) increased funding and allowed for flexibility in implementation of federal nutrition programs, including Child Nutrition Programs, the Special Supplemental Nutrition Program for Women, Infants and Children (WIC), and the Supplemental Nutrition Assistance Program (SNAP) [9]. Unfortunately, these measures are not enough to combat food insecurity, as the first published studies assessing food security during the Covid-19 pandemic reveal increases in food insecurity among rural [10] and adult populations with low income [11]. Feeding America, a nationwide network of hundreds of food banks, reported an increased demand for charitable food donations as affected individuals have been stocking up on food and other emergency supplies [12]. 

While many people are struggling financially during the pandemic, one population especially affected by Covid-19 is college students. Many college students will not be eligible for financial assistance during the Covid-19 pandemic. The Coronavirus Aid, Relief, and Economic Security (CARES) Act, funds the Higher Education Emergency Relief Fund, which provides funding to institutions for emergency financial aid to students and suspends student loan payments for six months. The amount of money students receive varies based on their financial need and the way the university allocates the funding [13]. One public university cites that eligible students will receive $1000 at most, and that online, international, and Deferred Action for Childhood Arrival (DACA) students are not eligible for these funds [14]. H.R. 133, the Consolidated Appropriations Act, signed into law on 27 December 2020, increased federal funding and expanded eligibility for nutrition assistance programs, including new eligibility requirements for college students who were impacted by Covid-19 with reduced work hours [15,16]. While these laws are helpful, they may not be enough to help universities and students recover from the pandemic, as evidenced by a statement from the Association of Public and Land Grant Universities stating, “the financial emergency facing colleges and universities demands so much more to stabilize institutions, support students, employees, and scientific research” [17]. College students may not have been eligible for other types of financial assistance like the Economic Impact Payments (EIPs), which provided individuals who met eligibility criteria with $1200 and then $600. If a parent claimed their college student as a dependent, then the student was not eligible for the payment themselves. Additionally, parents of college students were ineligible for additional money for their college student if the student was 17 years of age or older [18]. Despite these issues, future legislation may provide economic relief to college students, with two economic relief packages recently introduced in Congress with EIP inclusion guidelines for young adults [19,20].

Although the USDA has responded to the Covid-19 pandemic to help families access food for a healthy diet, many college students will not be eligible for food assistance through these channels. College students are only eligible for SNAP benefits if they are enrolled in college at least half time and meet at least one of the following criteria: 17 years old or younger or 50 years old or older, have a disability, receive Temporary Assistance for Needy Families (TANF), work at least 20 h, participate in various work study or on-the-job training programs, or care for a child [21]. A recent legal change may allow college students whose employment status was affected by the pandemic to now be eligible for SNAP benefits, though it is unclear how many more students will benefit from this expanded eligibility provision [16]. Some students who are eligible for SNAP benefits may not know about the program. A report from the US Government Accountability Office (GAO) published in 2018 urged FNS to improve dissemination of student eligibility information because almost 2 million potentially eligible college students did not report receiving benefits [22]. Legislation has been introduced to the House of Representatives (H.R.6756—End Pandemic Hunger for College Students Act of 2020 and H.R.6565—Emergency Ensuring Access to SNAP (EATS) Act of 2020) to ensure that college students are given access to emergency food assistance during the Covid-19 pandemic, but, unfortunately, action has not yet been taken [23,24]. 

Prior to Covid-19, college students were disproportionately affected by financial stress and food insecurity. This has been attributed to more students with low income going to college, higher college costs, insufficient student aid, a weak labor market for part-time workers, and SNAP policies that exclude many students from participation, among other reasons [25]. A 2017 systematic review found that food insecurity affected 32.9% of college students in the US (range: 14.1 to 58.8%), as reported in peer-reviewed literature [26]. A 2019 review found a wider range of food insecurity prevalence, 10–75% [27]. This is higher than the pre-Covid food insecurity rate for households in the US in 2019 of 10.5% [28]. Food insecurity rates are higher among certain college student demographic segments, including racial/ethnic minorities [26,29,30], younger students [26,29], international students [29], students with children [26], financially independent students [26], and Pell grant recipients [29]. Two reports by Laska et al. review proposed and enacted federal-level and state-level legislation related to college food insecurity [31,32]. During the 2019–2020 federal legislative session, 12 unique bills were introduced to address college food insecurity unrelated to the Covid-19 pandemic, though none were enacted [31]. States have had some success with passing legislation related to college food insecurity, with nine state-level laws, bills, or resolutions enacted in seven states to improve college food insecurity between 2017 and 2019 [32]. Covid-19 is likely making food insecurity issues worse for college students. A recent survey of 651 college students found that 34.5% of respondents experienced food insecurity within a 30-day period during the Covid-19 pandemic. Economic hardships such as being furloughed or laid off and experiencing a change in living situation were the strongest predictors of food insecurity [33]. 

While it has been well established that many students experience food insecurity during college and that the Covid-19 pandemic is creating a financial hardship for Americans, the literature is limited on changes in food security that college students are facing because of the Covid-19 pandemic. Therefore, the purpose of this study was to (i) identify and describe the prevalence of food insecurity at a public university before and after the onset of the Covid-19 pandemic and (ii) examine differences in academic and sociodemographic characteristics of students who experienced changes in food security as a result of Covid-19. 

## 2. Materials and Methods

### 2.1. Design and Participants

A cross-sectional, non-probability, web-based (Qualtrics) survey was completed by students attending a large, public land grant university. To improve survey validity, one validation question was included in the survey to address potential misreporting by participants. “Please answer ‘somewhat agree’ for this row. Thank you for reading carefully.” was placed within a question asking about feelings toward an on-campus food pantry. Participants who did not answer this question correctly were excluded from analysis. A sample of undergraduate and graduate students were asked to review the survey questions prior to survey distribution to ensure appropriate readability. The survey was distributed to all undergraduate and graduate students via email and was open for approximately 3.5 weeks between April 2020 and May 2020. This was several weeks after the onset of the Covid-19 pandemic and five weeks after the University transitioned students to online learning and postponed campus activities. Participants agreed to participate prior to taking the survey. Every third participant who completed the survey (up to the first 1200 students) received a $10 electronic gift card for participating. The study protocol received Institutional Review Board approval. 

### 2.2. Measures

#### 2.2.1. Food Insecurity

The ten-item US Adult Food Security Survey Module was adapted to assess the prevalence of food insecurity before the Covid-19 pandemic (pre-Covid) and after the onset of Covid-19 (post-Covid-onset) (Table 1) [34]. Eight questions (HH1, HH2, HH3, HH4, AD1, AD2, AD3, AD5) were asked twice within the following time frames: 1. “…in the past 12 months, prior to concerns about Covid-19” to assess food security status prior to the Covid-19 pandemic (pre-Covid) and 2. “…in the past few weeks” to assess food security after the onset of the Covid-19 pandemic (post-Covid-onset). Each question with two time frames was presented on the same screen so participants could see how they answered the question within the pre-Covid and post-Covid-onset time frames. Three questions (AD1a, AD4, and AD5a) were only asked once because they used predetermined time frames that were not modified for this study. The responses to AD1a, AD4, and AD5a questions were used in calculating pre-Covid and post-Covid-onset food security scores. 

Food security status was calculated by coding affirmative responses with a “1” and other responses with a “0” in accordance with the USDA Guide to Measuring Household Food Security to create raw composite scores for pre-Covid and post-Covid-onset time points [34]. Raw scores varied from 0–10 with higher scores indicating less food security. Pre-Covid raw scores were categorized into four food security categories: high food security (raw score 0), marginal food security (raw scores 1–2), low food security (raw scores 3–5), and very low food security (raw scores 6–10) for reporting purposes in Table 2.

Changes in food security status were calculated by comparing pre-Covid and post-Covid-onset raw scores. Participants were classified as less food secure at post-Covid-onset if their food security raw score was higher at the post-Covid-onset time point compared to the pre-Covid time point. Participants were classified as more food secure at the post-Covid-onset time point if their food security raw score was lower at the post-Covid-onset time point compared to the pre-Covid time point. Individuals who did not change food security status raw scores from pre-Covid to post-Covid-onset time points were categorized as no change in food security. 

This method of classifying changes in food security (i.e., change in raw score) was selected instead of measuring categorical changes (i.e., high, marginal, low, very low food security) to better account for acute changes in food security brought on after the onset of the Covid-19 pandemic. Some participants who were classified as becoming more or less food secure may have stayed within the same food security status category (high food security, marginal food security, low food security, very low food security). For example, a participant who received a raw score of 3 at the pre-Covid time point and raw score of 4 at the post-Covid-onset time point would be classified as becoming less food secure, though they remained in the “low food security” category. 

#### 2.2.2. Sociodemographic Variables

Participant characteristics, such as age, gender, ethnicity, race, living situation, university classification, residence status, employment status, Pell grant status, and financial status were collected. Additional questions were asked to assess changes in students’ sociodemographic characteristics since the Covid-19 outbreak, including, “Has your housing situation changed as a result of the Covid-19 outbreak?”, “Where are you currently living?” (if affirmative response to previous question), and “Has your employment status changed as a result of the Covid-19 outbreak?”.

### 2.3. Statistical Analysis

Descriptive statistics were calculated to describe the demographic characteristics as mean (standard deviation) for continuous variables and *n* (percentage) for categorical variables. Chi-square tests of independence were computed to determine if there were categorical associations between food security status and sociodemographic characteristics (Table 2) and between changes in food security status after the onset of Covid-19 and sociodemographic characteristics (Table 3). All expected cell frequencies were greater than five. Analyses were conducted in IBM SPSS Statistics for Macintosh, version 26 (Armonk, NY, USA). For statistical analysis, a *p*-value of <0.05 was considered statistically significant. 

## 3. Results

### 3.1. Study Population

Participants (*n* = 3206) were primarily female (70.7%), non-Hispanic (79.0%), White (72.9%), in-state students (83.9%) who resided off-campus not with parents pre-Covid (71.9%). A mix of students, ranging from freshman to graduate students participated in the survey. Just over half of the sample (54.0%) was employed at least part-time pre-Covid, and 65.2% received financial support from their family pre-Covid.

### 3.2. Pre-Covid Food Security Status

Pre-Covid food security status is displayed in Table 2. Prior to the Covid-19 pandemic, 56.1% (1797) of the students had high food security, 19.1% (613) had marginal food security, 12.2% (391) had low food security, and 12.6% (405) had very low food security. Pre-Covid food security status was significantly associated with gender (*p* = 0.007), race (*p* < 0.001), and ethnicity (*p* < 0.001). A greater proportion of students who identified as Hispanic/Latino experienced food insecurity compared to students who did not identify as Hispanic/Latino. Moreover, students who identified as Black/African American and other race/multiple races experienced food insecurity compared to students who identified as White or Asian. College student characteristics including university classification, residence status, and place of residence at the beginning of the Spring 2020 semester were significantly (*p* < 0.001) associated with pre-Covid food security status. Additionally, economic characteristics such as employment status at the beginning of the Spring 2020 semester, Pell grant status, and financial independence were significantly (*p* < 0.001) associated with pre-Covid food security status. Students who received a Pell grant and were financially independent experienced higher rates of food insecurity compared to other students in the sample. 

### 3.3. Changes in Food Security and Other Characteristics after the Onset of Covid-19 

Comparisons made in food security before and after the onset of Covid-19 were determined by comparing changes in food security survey raw scores. Approximately one in five students (22.6%) became less food secure after the onset of Covid-19, while 15% became more food secure. Change in food security raw score after the onset of the pandemic was significantly (*p* < 0.001) associated with pre-Covid food security status. Change in housing was significantly (*p* < 0.001) associated with change in food security status raw score after the onset of Covid-19. Over half (53.4%) of the sample experienced a change in housing situation, and a greater percentage of those who moved because of Covid-19 became more food secure compared to those who did not move (19.4% and 10.6%, respectively). Nearly all students reported living off-campus after the onset of Covid-19 (94.2%), and approximately half (50.7%) reported living with parents. When only looking at the subset of students who experienced a housing change because of Covid-19, 88.6% moved in with parents. A similar percentage of students who moved in with their parents became less food secure (17.9%) as those who became more food secure (19.9%). Pre-Covid employment status (*p* = 0.005) and change in employment status because of Covid-19 (*p* < 0.001) were both associated with change in food security status raw score after the onset of Covid-19. Changes in employment because of Covid-19 were common, with 19.7% of students experiencing a loss of employment. A higher proportion of students who were employed fewer hours (28.7%) or no longer employed (31.0%) became less food secure after the onset of Covid-19 compared to students employed more hours (25.8%) or whose employment status did not change (19.1%). Gender (*p* = 0.010), ethnicity (*p* < 0.001), and race (*p* < 0.001) were all associated with changes in food security raw score after the onset of Covid-19. Students who identified as Hispanic/Latino were less likely to retain the same food security status at the two time points compared to non-Hispanic/Latino students (52.1% and 64.6%, respectively). Over one quarter (28.1%) of Hispanic/Latino students became less food secure after the onset of Covid-19, and 19.7% became more food secure. Compared to other races, Asian students were more likely to become less food secure after the onset of Covid-19. Residence status was associated with change in food security raw score after the onset of Covid-19 (*p* < 0.001), with a greater proportion of international students (35.6%) becoming less food secure compared to in-state (22.2%) and out-of-state (19.1%) students. Additional characteristics associated with higher proportions of becoming less food secure after the onset of Covid-19 included being financially independent (*p* = 0.010) and being a Pell grant recipient (*p* < 0.001).

## 4. Discussion

The aim of this study was to assess the food security status of students at a large public university prior to and after the onset of the Covid-19 pandemic. Data show that 24.8% of the students experienced food insecurity prior to the pandemic, and that 38.0% of students experienced a change in food security after the onset of the pandemic. Of those who experienced a change (*n* = 1218), 59.6% became less food secure, and 40.4% became more food secure. 

One characteristic that was associated with a change in food security after the onset of Covid-19 was changing housing because of the pandemic. Over half of the participants (*n* = 1688, 53.4%) had a change in housing after the onset of Covid-19. Of those who did experience a change in housing, the majority (88.6%) moved in with their parents. Interestingly, moving home did not help guarantee increased access to food with similar percentages of students becoming more food secure (17.9%) and less food secure (19.9%). Students were encouraged to return home if possible, during an announcement made on 11 March 2020 [35]. Even though this decision was made to ensure student safety and reduce the transmission of Covid-19 on university campus, university administrators should consider the impact of such recommendations in the future since it may have impacted other factors that influence student health (i.e., food security). Making students aware of food assistance resources in their local communities, such as Feeding America’s local food bank finder [36], may help fill the gaps for students who frequently visit on-campus food pantries. 

Only 3.1% of students in the sample remained living on campus at the time the survey was administered. Approximately half of the students who remained on campus were undergraduate students (54.1%), while the remaining were graduate students (45.9%). When the university president recommended that students return home in the Spring 2020 semester, he also announced that resources would remain open for students who could not leave campus [35]. Dining halls and the on-campus food pantry continued to serve students on campus albeit with safety measures in place. Students, faculty, and staff using food pantry resources could order food online and identify a time for pick up, or they could stop by for a prepackaged bag of food. Despite these efforts, 29.6% of students who stayed on campus experienced a decrease in food security. This is slightly higher than the overall percentage of students who experienced a decrease in food security after the onset of Covid-19 (22.6%). It is unclear why students remained on campus, but one possible explanation is because of job requirements. The majority (71.4%) of students who remained on campus reported no change in their employment status after the onset of Covid-19, suggesting that they may have remained on campus to maintain employment. As such, it is important for university administrators to continue offering support to students who must live on campus during these situations. 

Like the housing situation, students also experienced changes in their employment status due to Covid-19. When asked how employment changed after the onset of the pandemic, 19.7% of respondents answered that they were no longer employed, and 9.2% said they were employed fewer hours. This parallels the spike in unemployment that was seen in the United States after the onset of Covid-19 [7]. And while the national unemployment rate has fallen since the largest increase seen in April 2020 [7], it is unclear how college students have managed to regain employment with college campuses, restaurants, entertainment venues, and other locations where college students may work not yet operating at full capacity. Additionally, with college students potentially ineligible for current Covid-19-specific economic relief [18] or federal food assistance [37], college students may not have the means to purchase adequate and nutritious food. The findings of this study align with reported findings from a cross-sectional survey administered by Owens et al., who reported that changing living arrangement and working fewer hours (via furlough, lay-offs, or reduced hours) predicted food insecurity among college students in Texas during the Covid-19 pandemic [32]. Data from this study show that 31% of students who lost their jobs because of Covid-19 became less food secure, which is a higher rate than the overall percentage of students who became less food secure after the onset of Covid-19 (22.6%).

Food insecurity during college is associated with poor academic outcomes [30], poor sleep quality [30], high stress [30], disordered eating behaviors [30], higher body mass index [38], less physical activity [38], poor dietary intake [38], and poor psychosocial health [39]. Furthermore, recent data reveal that the pandemic is affecting college students’ mental health status, including their stress, anxiety, and depressive thoughts [40]. While the long term academic, physical, and emotional health impacts of Covid-19 are not clear, the added negative effects of food insecurity can compound these issues making it especially detrimental for college students. College counseling, wellness, and financial affairs offices should be aware of the added stressors college students are facing and be prepared to support students during the pandemic. Lessons can be learned from institutions that developed support services for students before the onset of the Covid-19 pandemic. For example, the Single Stop U.S.A.’s Community College Initiative is a program designed to connect college students to financial and legal resources by supporting students with screening for public benefits, tax preparation, financial counseling, access to an attorney for legal services, and case management and referrals for wraparound support services [41]. A study of Single Stop found that students who used these services were more likely to continue into a second year of community college and take more credit hours compared to students who did not use the services [41]. Programs offered through colleges such as Single Stop are even more important during the Covid-19 pandemic, and professionals working for these programs should be prepared to support students with academic, financial, social, and health-related issues, especially as eligibility requirements and availability of public benefits are rapidly changing during the pandemic [42]. 

This study presents several strengths and limitations. The survey reached a large number of students (*n* = 3206). The timing of the dissemination of the survey and adaptation of the US Adult Food Security Survey Module questions allowed researchers to appreciate acute changes in food security that occurred just after the onset of the Covid-19 pandemic. Questions about changes in housing and employment status were added to the survey to better understand how college students were impacted by the pandemic. Finally, many demographic characteristics were collected to discover associations with food security before the pandemic and changes in food security after the onset of Covid-19. One limitation is the cross-sectional study design, which only provides a snapshot in time. Therefore, the researchers cannot assess changes in food security as the pandemic continued through the summer or attribute changes in food security specifically to the pandemic. The survey included questions that asked students to recall their food security situation currently (just after the onset of Covid-19) and over the past 12 months. Students may have experienced recall bias when answering these questions or other survey questions. Additionally, a convenience sample from one public university was used, which was not fully representative of all college students at the institution where the study was conducted, nor did authors post-stratify or weight the sample to account for this discrepancy.

## 5. Conclusions

Taken together, findings from this survey revealed that many college students experienced a shift in food security after the onset of Covid-19, which may be related to the closure of college campuses, changes in housing situation, or changes in employment. Because college students may be missing out on economic and nutrition assistance during the pandemic, it is important that policies are enacted at the university, community, state, and/or federal level to alleviate the financial and health burden that college students are facing during the pandemic. University administrators must be aware of the lack of federal- [31] and state-level [32] nutrition assistance for college students and find ways to ensure access to adequate amounts of nutritious food. Despite a majority of students leaving campus, a small number of students were unable to leave for various reasons. Universities must consider the needs of these students and continue providing safe housing and access to healthy food. Administrators should also remain up-to-date with current college food security legislation and make it a priority to assist students as relief becomes available. 

## Figures and Tables

**Table 1:** Food Security Survey Questions.

US Adult Food Security Survey Module Question Number	Pre-Covid Question	Post-Covid-Onset Question	Response Options with Scoring
HH2	“I worried whether my food would run out before I got money to buy more.” Was that often true, sometimes true in the past 12 months, prior to concerns about COVID-19?	“I worried whether my food would run out before I got money to buy more.” Was that often true, sometimes true in the past few weeks?	Often true (1) Sometimes true (1) Never true (0)
HH3	“The food that I bought just didn’t last, and I didn’t have the money to get more.” Was that often true, sometimes true, or never true for you in the past 12 months, prior to concerns about COVID-19?	“The food that I bought just didn’t last, and I didn’t have the money to get more.” Was that often true, sometimes true, or never true for you in the past few weeks?	Often true (1) Sometimes true (1) Never true (0)
HH4	“I couldn’t afford to eat balanced meals.” Was that often true, sometimes true, or never true for you in the past 12 months, prior to concerns about COVID-19?	“I couldn’t afford to eat balanced meals.” Was that often true, sometimes true, or never true for you in the past few weeks?	Often true (1) Sometimes true (1) Never true (0)
AD1	Did you ever cut the size of your meals or skip meals because there wasn’t enough money for food in the past 12 months, prior to concerns about COVID-19?	Did you ever cut the size of your meals or skip meals because there wasn’t enough money for food in the past few weeks?	Yes (1) No (0)
AD1a	How often did you cut the size or skip meals because there wasn’t enough money for food?		Almost every month (1) Some months, but not every month (1) Only 1 or 2 months (0)
AD2	Did you ever eat less than you felt you should because there wasn’t enough money for food in the past 12 months, prior to concerns about COVID-19?	Did you ever eat less than you felt you should because there wasn’t enough money for food in the past few weeks?	Yes (1) No (0)
AD3	Were you ever hungry but didn’t eat because there wasn’t enough money for food in the past 12 months, prior to concerns about COVID-19?	Were you ever hungry but didn’t eat because there wasn’t enough money for food in the past few weeks?	Yes (1) No (0)
AD4	In the last 12 months, did you lose weight because there wasn’t enough money for food?		Yes (1) No (0)
AD5	Did you ever not eat for a whole day because there wasn’t enough money for food in the past 12 months, prior to concerns about COVID-19	Did you ever not eat for a whole day because there wasn’t enough money for food in the past few weeks?	Yes (1) No (0)
AD5a	How often did you ever not eat for a whole day because there wasn’t enough money for food?		Almost every month (1) Some months, but not every month (1) Only 1 or 2 months (0)

Note: HH = Household Questions; AD = Adult Questions.

**Table 2:** Participant Sociodemographic Characteristics.

Characteristic	All Students (*n* = 3206) ^a^	High Food Security (Pre-Covid, *n* = 1797) ^b^	Marginal Food Security (Pre-Covid, *n* = 613) ^b^	Low Food Security (Pre-Covid, *n* = 391) ^b^	Very Low Food Security (Pre-Covid, *n* = 405) ^b^	*p*-Value
Age in Years, mean (SD)	22.5 (4.7)	22.5 (5.1)	22.5 (4.2)	22.4 (4.3)	22.4 (4.1)	
Gender, *n* (%)						0.007
Male	921 (29.3)	545 (59.2)	186 (20.2)	93 (10.1)	97 (10.5)	
Female	2217 (70.7)	1213 (54.7)	416 (18.8)	293 (13.2)	295 (13.3)	
Ethnicity, *n* (%)						<0.001
Hispanic/Latino	664 (21.0)	300 (45.2)	153 (23.0)	94 (14.2)	117 (17.6)	
Not Hispanic/Latino	2492 (79.0)	1465 (58.8)	453 (18.2)	294 (11.8)	280 (11.2)	
Race, *n* (%)						<0.001
White	2289 (72.9)	1331 (58.1)	446 (19.5)	263 (11.5)	249 (10.9)	
Black or African American	176 (5.6)	71 (40.3)	34 (19.3)	29 (16.5)	42 (23.9)	
Asian	439 (14.0)	245 (55.8)	87 (19.8)	57 (13.0)	50 (11.4)	
Other/multi-racial	236 (7.5)	115 (48.7)	34 (14.4)	34 (14.4)	53 (22.5)	
Live with Children, *n* (%)						0.447
Yes	129 (4.0)	76 (58.9)	28 (21.7)	14 (10.9)	11 (8.5)	
No	3077 (96.0)	1721 (55.9)	585 (19.0)	377 (12.3)	394 (12.8)	
University Classification, *n* (%)						<0.001
Freshman	492 (15.4)	305 (62.0)	81 (16.5)	49 (10.0)	57 (11.6)	
Sophomore	576 (18.0)	327 (56.8)	118 (20.5)	58 (10.1)	73 (12.7)	
Junior	541 (16.9)	287 (53.0)	96 (17.7)	79 (14.6)	79 (14.6)	
Senior	609 (19.0)	312 (51.2)	108 (17.7)	91 (14.9)	98 (16.1)	
Graduate student	987 (30.8)	565 (57.2)	210 (21.3)	114 (11.6)	98 (9.9)	
Residence Status, *n* (%)						<0.001
In-state	2657 (83.9)	1504 (56.6)	493 (18.6)	308 (11.6)	352 (13.2)	
Out-of-state	304 (9.6)	178 (58.6)	63 (20.7)	38 (12.5)	25 (8.2)	
International	205 (6.5)	90 (43.9)	51 (24.9)	43 (21.0)	21 (10.2)	
Place of Residence Spring 2020, *n* (%)						0.045
On-campus	677 (21.4)	397 (58.6)	130 (19.2)	71 (10.5)	79 (11.7)	
Off-campus, Not with Parents/Guardians	2276 (71.9)	1235 (54.3)	447 (19.6)	300 (13.2)	294 (12.9)	
Off-campus, with Parents/Guardians	116 (3.7)	75 (64.7)	15 (12.9)	11 (9.5)	15 (12.9)	
Other	95 (3.0)	65 (68.4)	15 (15.8)	7 (7.4)	8 (8.4)	
Employment Status Spring 2020, *n* (%)						<0.001
Employed Full-time	322 (10.2)	185 (57.5)	65 (20.2)	37 (11.5)	35 (10.9)	
Employed Part-time (20–29 h)	521 (16.5)	258 (49.5)	105 (20.2)	78 (15.0)	80 (15.4)	
Employed Part-time (1–19 h)	861 (27.3)	439 (51.0)	165 (19.2)	119 (13.8)	138 (16.0)	
Not Employed	1452 (46.0)	884 (60.9)	271 (18.7)	153 (10.5)	144 (9.9)	
Pell Grant Recipient, *n* (%)						<0.001
Yes	695 (22.0)	276 (39.7)	148 (21.3)	114 (16.4)	157 (22.6)	
No	2470 (78.0)	1496 (60.6)	459 (18.6)	274 (11.1)	241 (9.8)	
Receive Financial Support from Family, *n* (%)						<0.001
Yes	2054 (65.2)	1252 (61.0)	368 (17.9)	232 (11.3)	202 (9.8)	
No	1096 (34.8)	511 (46.6)	238 (21.7)	155 (14.1)	192 (17.5)	
Financially Independent, *n* (%)						<0.001
Yes	1172 (37.2)	573 (48.9)	252 (21.5)	158 (13.5)	189 (16.1)	
No	1977 (62.8)	1189 (60.1)	354 (17.9)	229 (11.6)	205 (10.4)	

^a^ Numbers in parentheses indicate percentages for column. ^b^ Numbers in parentheses indicate percentages for row. Note: SD = Standard Deviation.

**Table 3:** Participant Characteristics and Associations with Changes in Food Security during Covid-19.

Characteristic	All Students (*n* = 3206) ^a^	Less Food Secure after the Onset of Covid-19 (i.e., Increase in Food Security Raw Score) (*n* = 726) ^b^	No Change in Food Security after the Onset of Covid-19 (*n* = 1988) ^b^	More Food Secure after the Onset of Covid-19 (i.e., Decrease in Food Security Raw Score) (*n* = 492) ^b^	*p*-Value
Food Security Characteristics					
Pre-Covid Food Security Status, *n* (%)					<0.001
High Food Security	1797 (56.1)	320 (17.8)	1477 (82.2)	0 (0.0)	
Marginal Food Security	613 (19.1)	202 (33.0)	255 (41.6)	156 (25.4)	
Low Food Security	391 (12.2)	130 (33.2)	102 (26.1)	159 (40.7)	
Very Low Food Security	405 (12.6)	74 (18.3)	154 (38.0)	177 (43.7)	
Housing Characteristics					
Change in Housing Situation Because of Covid-19, *n* (%)					<0.001
Yes	1688 (53.4)	321 (19.0)	1039 (61.6)	328 (19.4)	
No	1475 (46.6)	397 (26.9)	922 (62.5)	156 (10.6)	
Housing During Covid-19 Among All Students, *n* (%)					<0.001
On-Campus Housing	98 (3.1)	29 (29.6)	60 (61.2)	9 (9.2)	
Off-Campus Housing, Not with Parents	1374 (43.5)	379 (27.6)	841 (61.2)	154 (11.2)	
Off-Campus Housing, with Parents	1603 (50.7)	293 (18.3)	1003 (62.6)	307 (19.2)	
Other	87 (2.8)	16 (18.4)	57 (65.5)	14 (16.1)	
Housing During Covid-19 Among Students Who Experienced a Housing Change, *n* (%)					0.011
Off-Campus Housing, Not with Parents	129 (7.6)	39 (30.2)	68 (52.7)	22 (17.1)	
Off-Campus Housing, with Parents	1495 (88.6)	268 (17.9)	930 (62.2)	297 (19.9)	
Other	64 (3.8)	14 (21.9)	41 (64.1)	9 (14.1)	
Live with Children, *n* (%)					0.026
Yes	129 (4.0)	33 (25.6)	87 (67.4)	9 (7.0)	
No	3077 (96.0)	693 (22.5)	1901 (61.8)	483 (15.7)	
Employment Characteristics					
Pre-Covid Employment Status During Spring 2020, *n* (%)					0.005
Employed Full-Time	322 (10.2)	74 (23.0)	205 (63.7)	43 (13.4)	
Employed Part-Time (20–29 Hours)	521 (16.5)	149 (28.6)	289 (55.5)	83 (15.9)	
Employed Part-Time (1–19 Hours)	861 (27.3)	200 (23.2)	524 (60.9)	137 (15.9)	
Not Currently Employed	1452 (46.0)	294 (20.2)	938 (64.6)	220 (15.2)	
Change in Employment Status Due to Covid-19, *n* (%)					<0.001
Employed Less Hours	279 (9.2)	80 (28.7)	149 (53.4)	50 (17.9)	
No Longer Employed	597 (19.7)	185 (31.0)	306 (51.3)	106 (17.8)	
Employed More Hours	93 (3.1)	24 (25.8)	58 (62.4)	11 (11.8)	
Has Not Changed	2055 (68.0)	392 (19.1)	1364 (66.4)	299 (14.5)	
Other Demographic Characteristics					
Gender, *n* (%)					0.010
Male	921 (29.3)	214 (23.2)	594 (64.5)	113 (12.3)	
Female	2217 (70.7)	498 (22.5)	1352 (61.0)	367 (16.6)	
Ethnicity, *n* (%)					<0.001
Hispanic/Latino	664 (21.0)	187 (28.2)	346 (52.1)	131 (19.7)	
Not Hispanic/Latino	2492 (79.0)	530 (21.3)	1609 (64.6)	353 (14.2)	
Race, *n* (%)					<0.001
White	2289 (72.9)	499 (21.8)	1453 (63.5)	337 (14.7)	
Black or African American	176 (5.6)	37 (21.0)	104 (59.1)	35 (19.9)	
Asian	439 (14.0)	134 (30.5)	256 (58.3)	49 (11.2)	
Other/Multi-Racial	236 (7.5)	44 (18.6)	136 (57.6)	56 (23.7)	
Student Classification, *n* (%)					0.003
Freshman	492 (15.4)	92 (18.7)	307 (62.4)	93 (18.9)	
Sophomore	576 (18.0)	141 (24.5)	341 (59.2)	94 (16.3)	
Junior	541 (16.9)	119 (22.0)	327 (60.4)	95 (17.6)	
Senior	609 (19.0)	151 (24.8)	364 (59.8)	94 (15.4)	
Graduate Student	987 (30.8)	223 (22.6)	648 (65.7)	116 (11.8)	
Residence Status, *n* (%)					<0.001
In-State	2657 (83.9)	589 (22.2)	1660 (62.5)	408 (15.4)	
Out-Of-State	304 (9.6)	58 (19.1)	199 (65.5)	47 (15.5)	
International	205 (6.5)	73 (35.6)	103 (50.2)	29 (14.1)	
Financially Independent, *n* (%)					0.010
Yes	1172 (37.2)	296 (25.3)	688 (58.7)	188 (16.0)	
No	1977 (62.8)	419 (21.2)	1264 (63.9)	294 (14.9)	
Pell Grant Recipient, *n* (%)					<0.001
Yes	695 (22.0)	206 (29.6)	364 (52.4)	125 (18.0)	
No	2470 (78.0)	514 (20.8)	1597 (64.7)	359 (14.5)	

^a^ Numbers in parentheses indicate percentages for column. ^b^ Numbers in parentheses indicate percentages for row.

## Data Availability

The data presented in this study are available upon request by the corresponding author.
